# An interpretable machine learning model for predicting depression in middle-aged and elderly cancer patients in China: a study based on the CHARLS cohort

**DOI:** 10.1186/s12888-025-07074-x

**Published:** 2025-07-01

**Authors:** Yue Xiao, Zejin Zhao, Chen-guang Su, Jian Li, Jinlong Liu

**Affiliations:** 1 Department of Hepatobiliary Surgery, The Affiliated Hospital of Chengde Medical University, Chengde, 067000 Hebei Province China; 2 Hebei Key Laboratory of Panvascular Diseases, Chengde, 067000 Hebei Province China

**Keywords:** Depression, Machine learning, CHARLS, SHAP, Shapley additive explanation, Cancer

## Abstract

**Background:**

Depression is very common in middle-aged and elderly cancer patients, which will seriously damage the quality of life and treatment effect of patients. This study aims to use machine learning methods to develop a predictive model to identify depression risk. However, since the traditional machine learning models have ‘black box nature’, Shapley Additive exPlanation is used to determine the key risk factors.

**Methods:**

This study included 743 cancer patients aged 45 and above from the 2011–2020 China Health and Retirement Longitudinal Study (CHARLS), and analyzed a total of 19 variables including demographic factors, economic factors, health factors, family factors, and personal factors. After screening the predictive features by LASSO regression, in order to determine the best model for prediction, six machine learning models—Support Vector Machine, K-Nearest Neighbors, Multi-layer Perceptron, Decision Tree, XGBoost and Random Forest were trained.

**Results:**

After training, the random forest model showed the best decision performance, AUC (95% CI): 0.774 (0.740–0.809). Subsequently, the model was interpreted by Shapley Additive exPlanation, and five key characteristics affecting the risk of depression were found. The feature importance plot intuitively shows that the predicted depression risk is significantly increased for patients with poor life satisfaction.

**Conclusions:**

We developed a clinical visualization model for health care providers to estimate the risk of depression in middle-aged and elderly cancer patients. As a powerful tool for early identification of depressive symptoms in middle-aged and elderly cancer patients, this model enables medical workers to implement clinical interventions earlier to obtain better clinical benefits.

**Supplementary Information:**

The online version contains supplementary material available at 10.1186/s12888-025-07074-x.

## Introduction

Cancer remains one of the most formidable global health challenges in the 21st century. It encompasses multiple different systems, characterized by the uncontrolled growth and spread of abnormal cells, which invade the surrounding tissues and organs, causing severe impairment of their functions [1]. In 2020 alone, an estimated 19.3 million new cancer cases have been diagnosed and about 10 million cancer-related deaths have occurred [2]. Although the current medical security and support system for cancer patients has been relatively well-established, the psychological health factors of cancer patients have still been long neglected. A meta-analysis found that, in China, the pooled prevalence of depression among cancer patients is as high as 45%, which will pose a significant threat to the quality of life of cancer patients [3]. Compared with the high risk of getting sick, the recognition rate and treatment rate of cancer-related depression among Chinese patients are extremely low [4]. Therefore, the effective identification of depression in cancer patients is particularly important. However, the high cost of conducting large-scale surveys greatly reduces the possibility of implementation.

Recently, machine learning (ML) has been widely applied in the medical field, especially making significant contributions to the early identification of diseases [5]. ML, as a crucial branch in the field of computer science, has gradually emerged as a powerful tool for predictive disease modeling [6]. Its core objective lies in achieving the optimization and improvement of various tasks by deeply mining and extracting the latent patterns within the data [7].

Although previous studies have used ML algorithms to construct a prediction model for depressive symptoms in patients with advanced breast cancer [8], the inherent ‘black box nature’ of traditional ML seriously restrict its clinical application [9]. Interpretable ML, which is derived from conventional ML, uses the Shapley Additive exPlanation (SHAP) based on cooperative game theory [10]. By combining SHAP, it effectively overcomes the defect of poor interpretability inherent in traditional ML models. Specifically, SHAP quantifies the contribution of each input feature to identify the key determinants of depression risk in middle-aged and elderly cancer patients. In addition, SHAP also provides a series of intuitive visualization tools, such as summary plot, dependency plot, and force plot, which transform complex model outputs into intuitive insights, thereby promoting model-to-clinical transformation.

As far as we know, this study is the first to use interpretable ML method to predict the depression risk of middle-aged and elderly cancer patients. By leveraging the large-scale dataset of the China Health and Retirement Longitudinal Study (CHARLS), the study aims to thoroughly explore the relationship between middle-aged and elderly cancer patients in China and depression, analyze the risk factors that trigger depression, and thus provide targeted and individualized strategies for the management and prevention of cancer patients in the future.

## Method

### Data sources and study design

The data used in this study are from the CHARLS database. CHARLS is a large-scale, multi-center, prospective longitudinal cohort study, which covers relevant data such as the health status, economic levels, and demographic factors of middle-aged and elderly people in 28 provincial administrative regions, 150 counties, and 450 villages across China [11]. The baseline survey was completed in 2011–2012, followed by a follow-up every 2 to 3 years. The research procedure of CHARLS strictly followed the Helsinki Declaration and was approved by the Ethics Committee of Peking University (Approval No. IRB00001052-11015). All respondents signed written informed consent before entering the group.

**Fig. 1:**
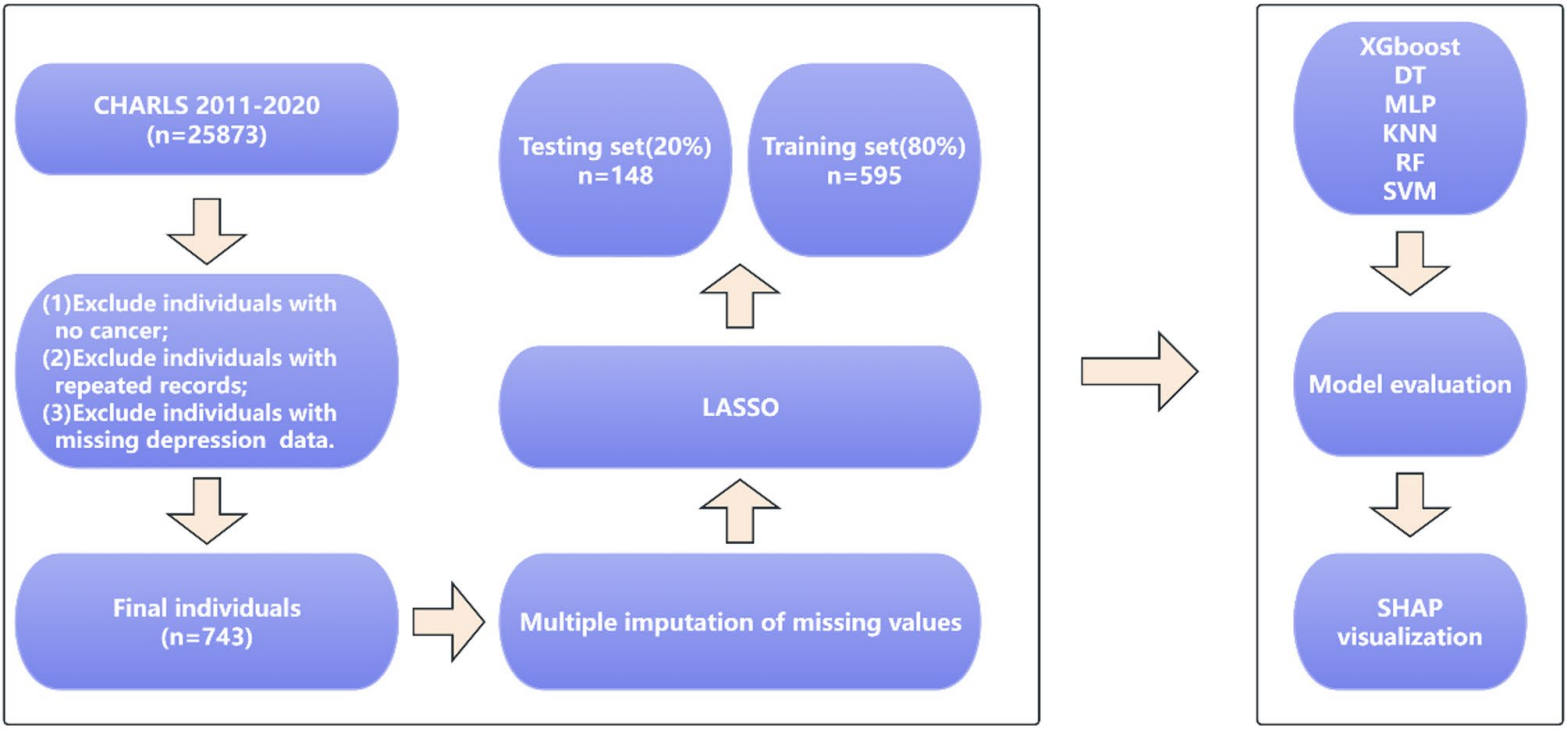
Research flow chart

This study used data from a total of five follow-up cycles from 2011 to 2020, and the final participants were determined by the following criteria: (1) Individuals with any cancer; (2) Exclude individuals with missing depression follow-up data; (3) Exclude individuals aged < 45 years. After a rigorous screening process, a total of 743 individuals were used for model construction. The screening process of the participants and the research design diagram are shown in Fig. 1.

### Feature selection

Based on previous research and related experience [12, 13], we selected the following 19 candidate predictors that may be associated with depression, including demographic characteristics (age, gender, education level, marital status), family characteristics (family size, parents’ economic support for children, children’s economic support for parents, health insurance), personal basic situation questionnaire (life satisfaction situation, social situation), health factors (history of falls, comorbidities, history of pain, smoking, drinking, self-rated health, average sleep time, ADL score, IADL score). The level of education is classified as illiteracy and non-illiteracy. Life satisfaction, self-health evaluation, ADL and IADL were investigated by questionnaire, and the results were expressed as scores.

In addition, the effective variables are screened by the least absolute shrinkage and selection operator (LASSO) regression algorithm. LASSO regression can change the smaller weight in the coefficient vector to 0 by introducing L1 regularization (i.e., LASSO penalty term). By selecting the features corresponding to the non-zero coefficients, the features with the greatest predictive ability for the target variables can be screened out, thereby obtaining a more refined model [14].

### Evaluation of outcome variables

In this study, we are interested in the outcome of the occurrence of depressive symptoms. The CESD-10 scale was used to assess depressive symptoms. Studies have shown that the scale has shown strong efficacy in the detection of depression in Chinese adults [15]. The CESD-10 scale consists of 10 items, each item is scored according to the level of 0 to 3, 0 represents “none”, and 3 represents “almost every day”, covering different frequency of symptoms. The total score of the scale ranged from 0 to 30 points, and the score was positively correlated with the severity of depressive symptoms, that is, the higher the score, the more severe the depressive symptoms. When the score reached 12 points or more, it was judged as a positive result.

### Missing value processing

Missing data is very common in the CHARLS database. Directly excluding individuals with missing variables may lead to sample representativeness bias, which affects the universality and accuracy of research conclusions. Therefore, we excluded variables with a missing value rate greater than 20% and used the Mice package in R software to perform multiple imputations on the remaining variables. The lack of data before interpolation is shown in Supplementary Fig. 1.

### Model construction

We developed six ML models, namely Support Vector Machine (SVM), K-Nearest Neighbor (KNN), Decision Tree (DT), Random Forest (RF), Multilayer Perceptron (MLP) and EXtreme Gradient Boosting (XGBoost). SVM is a powerful and versatile supervised learning model, which performs well in handling complex datasets [16]. KNN is an instance-based learning algorithm that is insensitive to outliers. Even when there is noise in the data or individual abnormal data points exist, it can still maintain relatively stable performance [17]. The working principle of DT is to use feature values to split the dataset into more manageable subgroups. Each internal node represents an attribute test, each branch represents the test result, and each leaf node represents a class label (decision) [18]. The RF algorithm is one of the most commonly used ensemble learning techniques for regression and classification. It constructs multiple decision trees by randomly selecting samples and combines them to provide more reliable and accurate predictions [19]. MLP is a feed-forward neural network. Its advantages lie in its strong non-linear mapping ability and good scalability, enabling it to flexibly deal with data of different scales and complexities [20]. XGBoost is an algorithm based on Gradient Boosting Decision Tree (GBDT), with strong generalization ability and excellent performance in various data mining and ML competitions [21]. In order to accurately evaluate the performance and generalization ability of the prediction model, we randomly divide the data set. Specifically, the data set is divided into a ratio of 80:20, with 80% of the training set and 20% of the test set. The training of the prediction model is completed in the training set, and the testing set is used to test the prediction model.Synthetic minority oversampling technique (SMOTE) was used to solve the class imbalance problem that may exist in the result variables, and all models used a 10-fold cross-validation method during training. For each ML algorithm, hyperparameter optimization was performed to maximize predictive performance. The strategies were as follows: (1) Grid search was applied to DT, XGBoost, RF, and SVM for systematic parameter exploration. (2) Bayesian optimization was employed for MLP to efficiently navigate its high-dimensional parameter space. (3) Random search was used for KNN to balance optimization efficacy.

### Statistical analysis

Continuous variables are presented as mean ± standard deviation (SD), and categorical variables are presented as frequency (%). Continuous variables conforming to normal distribution were compared between groups using independent t-tests, while continuous variables not conforming to normal distribution were compared using Mann-Whitney U test. The Chi-square test was used for the comparison of categorical variables between groups. For the coding of binary variables, ‘1’ corresponds to ‘yes’, while ‘0’ corresponds to ‘no’. Subsequently, evaluation indicators were constructed to evaluate the performance of the model, including AUC area and 95% confidence interval (CI), sensitivity, specificity, accuracy and 95% CI. Calibration curves were applied to assess the accuracy of the model’s probability predictions. Finally, in view of the black box nature of the ML models, SHAP values were adopted to visualize the prediction results. All statistical analyses were conducted using R software (Version 4.4.1; R Foundation for Statistical Computing, Vienna, Austria). A two-sided *P*-value < 0.05 was considered to indicate statistical significance.

## Result

### Subject characteristics

A total of 743 participants were included in the ML models to predict depression in cancer patients, among whom 293 (39.43%) were diagnosed with depression (Table 1). Patients with depression were more likely to be female, uneducated, unmarried, have a history of falls, comorbidities, and pain, participate less in social activities, have a lower average self-rated health score, lower average life satisfaction, shorter average sleep duration, and higher average IADL and average ADL scores.

**Table 1:** Baseline characteristics of the participants

	Total	Non- depression	Depression	*p*. overall
	*N* = 743	*N* = 450	*N* = 293	
Age	61.2 (9.28)	61.1 (9.55)	61.3 (8.87)	0.778
Gender				**0.012**
Female	470 (63.3%)	268 (59.6%)	202 (68.9%)	
Male	273 (36.7%)	182 (40.4%)	91 (31.1%)	
Education				**<0.001**
Illiteracy	315 (42.4%)	161 (35.8%)	154 (52.6%)	
Non-illiteracy	428 (57.6%)	289 (64.2%)	139 (47.4%)	
Marry				**0.003**
No	84 (11.3%)	38 (8.44%)	46 (15.7%)	
Yes	659 (88.7%)	412 (91.6%)	247 (84.3%)	
Fall-down				**0.004**
No	614 (82.6%)	387 (86.0%)	227 (77.5%)	
Yes	129 (17.4%)	63 (14.0%)	66 (22.5%)	
Insurance				0.122
No	27 (3.63%)	12 (2.67%)	15 (5.12%)	
Yes	716 (96.4%)	438 (97.3%)	278 (94.9%)	
Comorbidities				**<0.001**
No	195 (26.2%)	140 (31.1%)	55 (18.8%)	
Yes	548 (73.8%)	310 (68.9%)	238 (81.2%)	
Drink				0.127
No	581 (78.2%)	343 (76.2%)	238 (81.2%)	
Yes	162 (21.8%)	107 (23.8%)	55 (18.8%)	
Smoke				0.698
No	487 (65.5%)	292 (64.9%)	195 (66.6%)	
Yes	256 (34.5%)	158 (35.1%)	98 (33.4%)	
Pain				**<0.001**
No	311 (41.9%)	232 (51.6%)	79 (27.0%)	
Yes	432 (58.1%)	218 (48.4%)	214 (73.0%)	
Social				**0.029**
No	355 (47.8%)	200 (44.4%)	155 (52.9%)	
Yes	388 (52.2%)	250 (55.6%)	138 (47.1%)	
Srh	2.39 (1.00)	2.64 (1.00)	1.99 (0.86)	**<0.001**
Family size	3.07 (1.61)	3.11 (1.56)	3.02 (1.69)	0.468
Cfsfp	6547 (16245)	7100 (17887)	5698 (13321)	0.222
Pfsfc	4777 (39023)	4123 (16575)	5783 (58698)	0.637
Satlife	3.13 (0.81)	3.34 (0.67)	2.79 (0.90)	**<0.001**
Sleep time	5.91 (1.92)	6.35 (1.76)	5.25 (1.97)	**<0.001**
IADL	0.71 (1.22)	0.41 (0.93)	1.17 (1.44)	**<0.001**
ADL	0.59 (1.19)	0.37 (0.94)	0.93 (1.42)	**<0.001**

Srh: Self-rated health; Cfsfp: Children’s financial support for parents; Pfsfc: Parents’ financial support for their children; Satlife: life satisfaction score; IADL: Instrumental Activity of Daily Living; ADL: Activity of Daily Living

### Variable selection

**Fig. 2:**
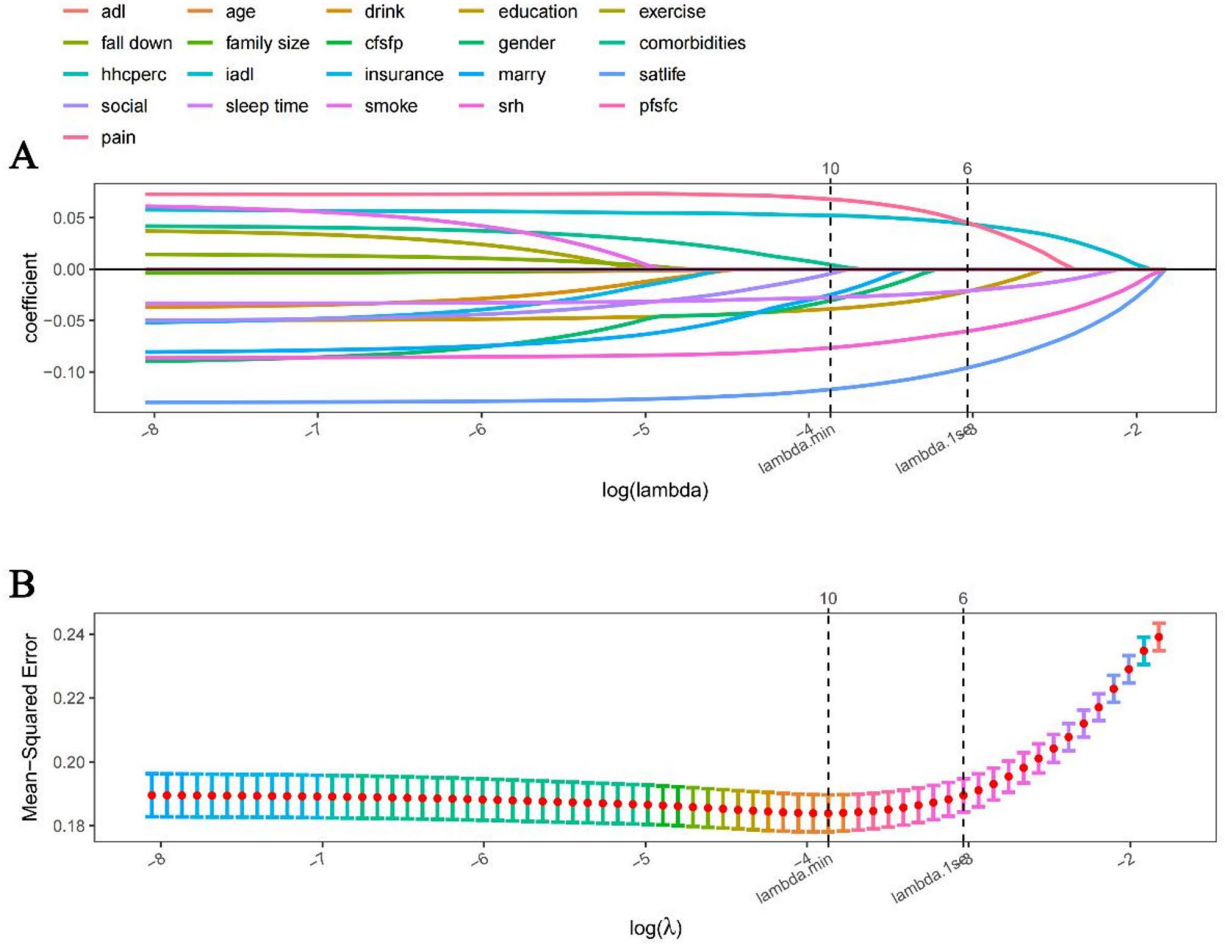
The results of the LASSO regression analysis. (**A**) The process of selection. (**B**) Average deviation and confidence interval

The LASSO regression was used to reduce the number of factors. The 10-fold cross-validation method was used for iterative analysis. The screening process is shown in Fig. 2A. Each curve represents the change trajectory of different variables, and the more important the variable is, the later it approaches 0. In the graph depicted in Fig. 2B, two conspicuous dashed lines stand out prominently. These lines respectively denote two distinct and significant λ values, namely λ_min_ and λ_1se_. When it comes to practical applications, any λ value situated within the interval between λ_min_ and λ_1se_ is deemed to fall within a reasonable range for the model, providing a balanced and suitable parameter setting for the model’s performance. λ_min_ is the minimum cross-validation error value of λ, and λ_1se_ is an estimated value obtained by considering the complexity and stability of the model on the basis of λ_min_. Considering the cross-validation error value and in order to retain as many features as possible, the fitting result of λ_min_ is selected. Ultimately, 10 variables were included, including gender, marital status, self-rated health, life satisfaction score, sleep time, education level, IADL, comorbidities, pain history and social activities.

### Model evaluation

**Fig. 3:**
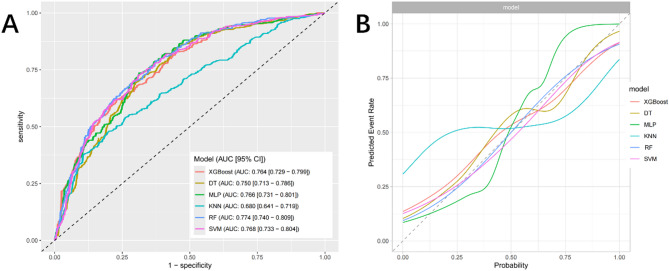
ROC curves and calibration curves for the machine learning models. (**A**) ROC curves. (**B**) Calibration curves. AUC: area under the curve; CI: confidence interval; XGBoost: EXtreme Gradient Boosting; DT: Decision Tree; MLP: Multilayer Perceptron; KNN: K-Nearest Neighbors; RF: Random Forest; SVM: Support Vector Machine

The discriminant ability of the six models was evaluated in the training set. Except for the KNN model, the other five models showed strong predictive performance, among which the RF model had the highest performance (AUC = 0.774, 95% CI: 0.740–0.809) (Fig. 3A). In addition, Table 2 also shows the overall performance of different prediction models. The RF model still shows superior overall performance (sensitivity: 0.802, specificity: 0.828). However, interestingly, the MLP model demonstrated higher accuracy (0.730), with the RF model closely following behind (0.724). Subsequently, to evaluate the calibration degree of the ML model intuitively and accurately, we also plotted the calibration curves of different models (Fig. 3B), and the RF model stood out among the six models. Obviously, the RF algorithm can be used as a powerful tool for depression prediction. Therefore, the RF model is included in subsequent studies.

**Table 2:** Performance metrics for the models

Model	AUC (95% CI)	Sensitivity	Specificity	Accuracy (95% CI)
XGBoost	0.764 (0.729–0.799)	0.772	0.619	0.714 (0.680–0.747)
DT	0.750 (0.713–0.786)	0.550	0.806	0.722 (0.693–0.759)
MLP	0.766 (0.731–0.801)	0.689	0.736	0.730 (0.696–0.761)
KNN	0.680 (0.641–0.719)	0.754	0.474	0.635 (0.599–0.670)
RF	0.774 (0.740–0.809)	0.802	0.828	0.724 (0.690–0.756)
SVM	0.768 (0.733–0.804)	0.689	0.729	0.716 (0.682–0.748)

AUC: area under the curve; CI: confidence interval; XGBoost: EXtreme Gradient Boosting; DT: Decision Tree; MLP Multilayer Perceptron; KNN: K-Nearest Neighbors; RF: Random Forest; SVM Support Vector Machine

### Feature importance ranking based on random forest model

**Fig. 4:**
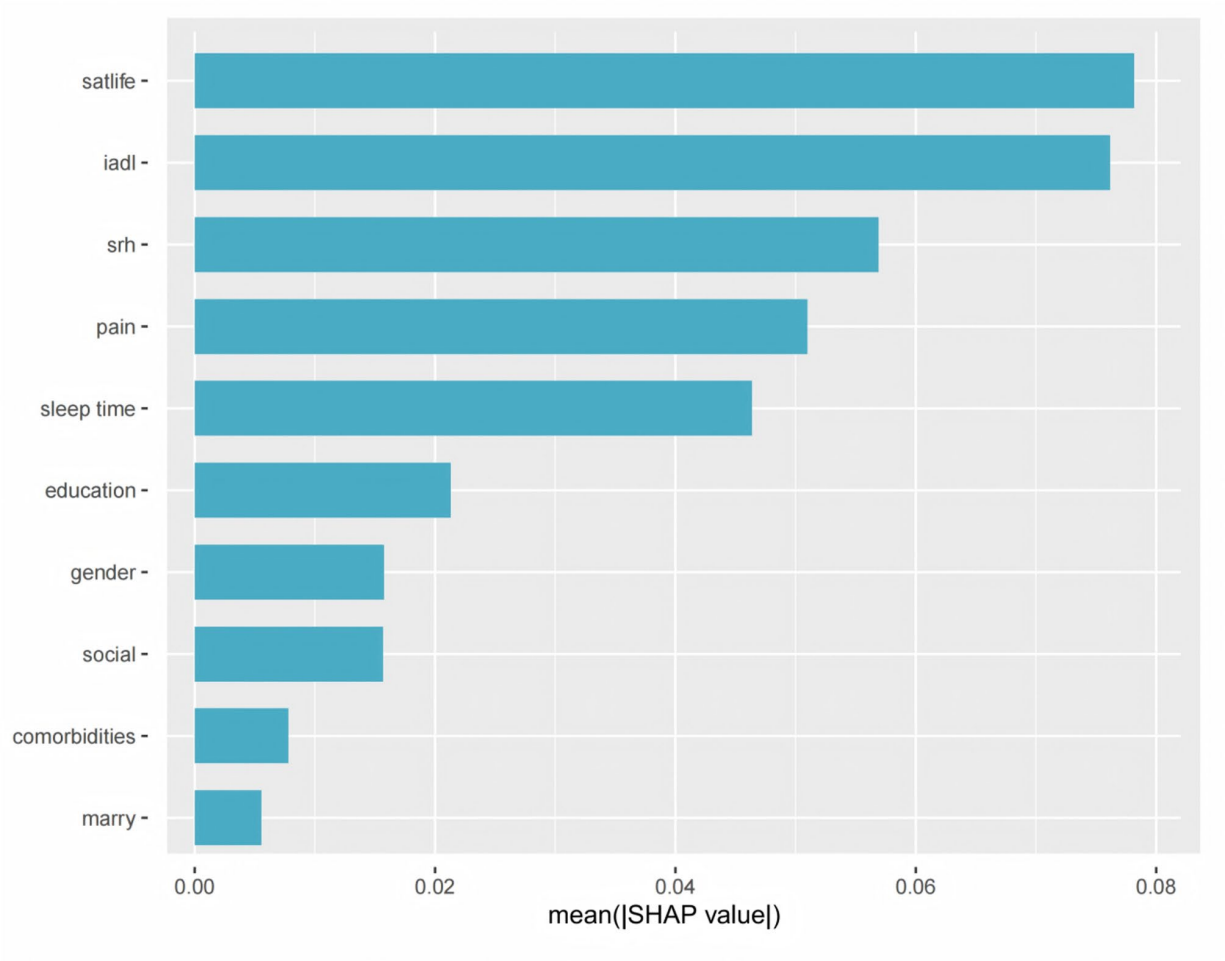
SHAP feature importance plot. SHAP feature importance is illustrated by the mean absolute SHAP value of each feature

To evaluate the roles of different predictive factors, we calculated the feature importance of the RF model (Fig. 4). Sorted according to the average absolute SHAP values from high to low, the feature importance gradually decreases from top to bottom along the y-axis. The top five risk features include self-rated health score, IADL, life satisfaction score, history of pain, and sleep time.

**Fig. 5:**
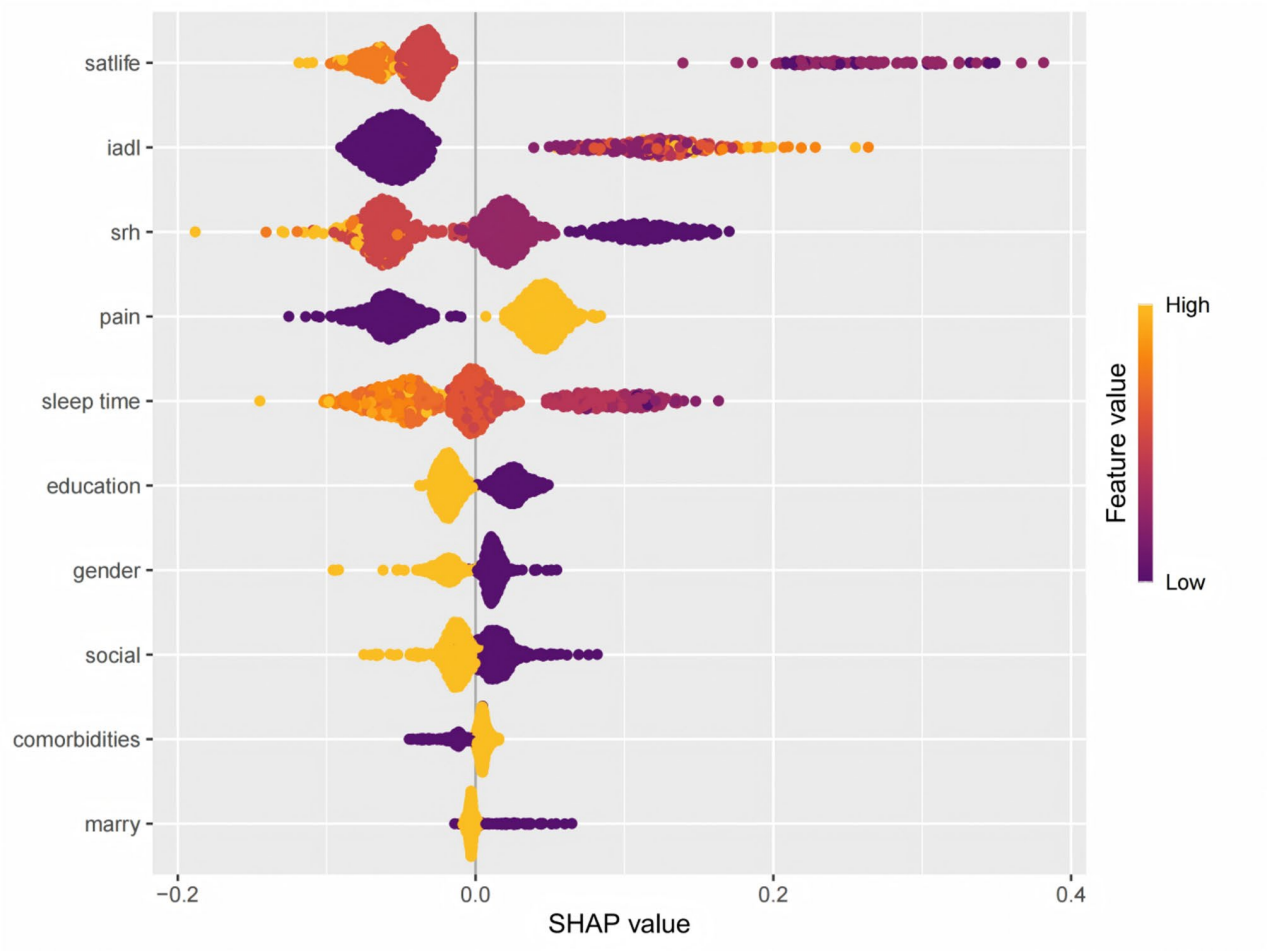
SHAP summary plot

**Fig. 6:**
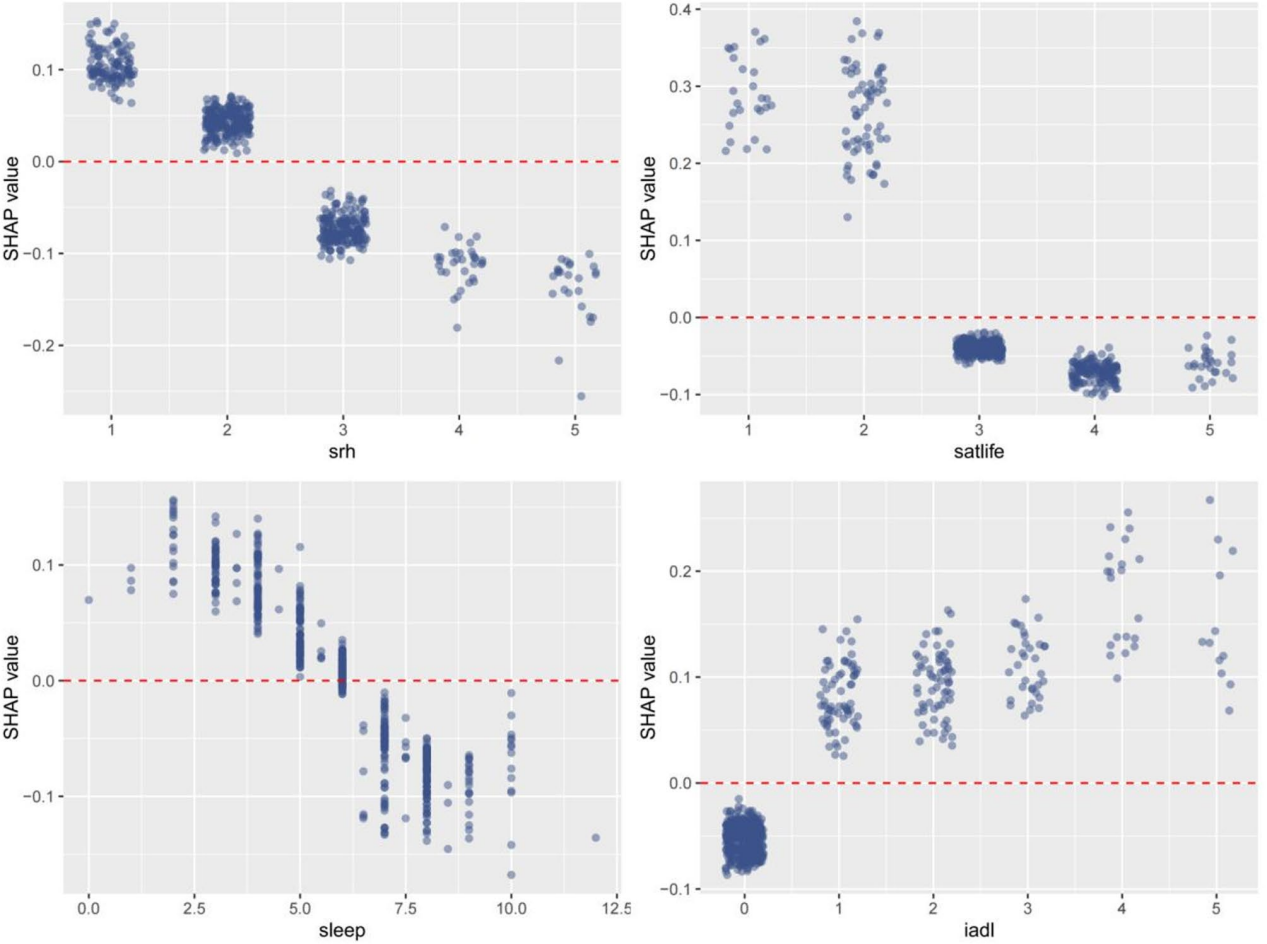
SHAP dependence plots

Figure 5 presents a summary plot that intuitively illustrates the impact of variables on the prediction. Yellow represents the high values of the variables, while purple represents the low values of the variables. For example, a higher life satisfaction score is associated with a negative SHAP value, indicating that individuals are more likely to be free from the risk of depression. Similarly, a higher IADL score suggests a greater likelihood of being threatened by depression. The SHAP dependence plot in Fig. 6 further clarifies the influence of each analyzed variable on the prediction of the RF model. Specifically, as the self-rated health score, life satisfaction score, and sleep duration increase, the SHAP values correspondingly rise. Conversely, an increase in the IADL score leads to a decrease in the SHAP value.

### The clinical application of models

**Fig. 7:**
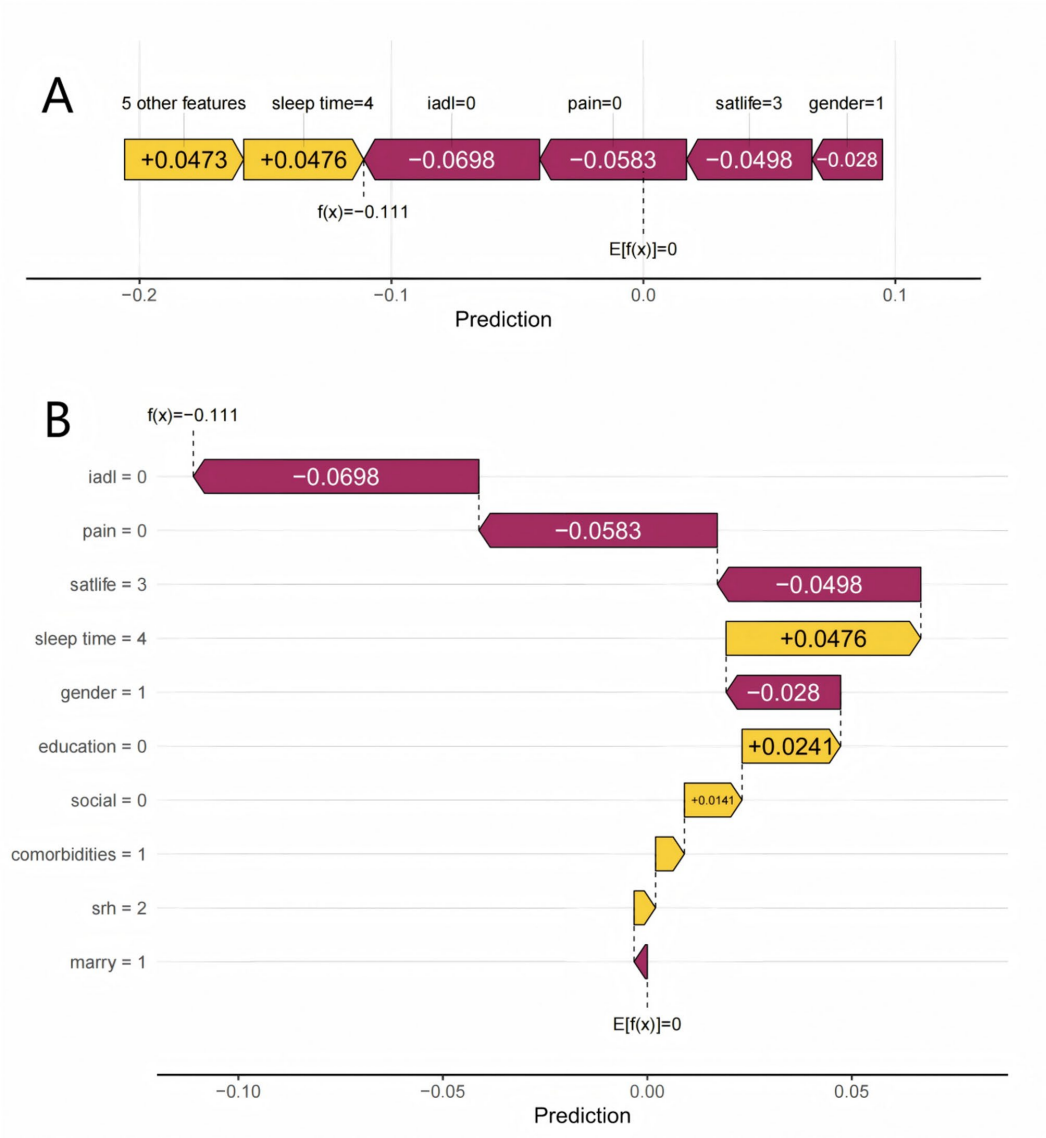
SHAP individual prediction visualizations plots. (**A**) Force plot. (**B**) Waterfall plot

To further explore the model’s prediction process for specific patients, we randomly selected an individual and plotted a visualization graph. In this visualization (Fig. 7A), yellow indicates the promotion of the development of depression, while purple indicates the hindrance of the development of depression. For this patient, the risk of depression predicted by the RF model was lower than the baseline. Among them, the top three contributing factors were IADL score of 5 points, no history of pain, and life satisfaction score of 3 points. The remaining risk factors affecting the RF model prediction are shown in the waterfall diagram (Fig. 7B).

### Correlation matrix of variables

Supplementary Fig. 2 shows the correlations between various variables. The IADL score is negatively correlated with sleep duration and self-rated health score. The life satisfaction rating is positively correlated with the self-rated health score. The IADL score and comorbidities are positively correlated with pain. Sleep duration is positively correlated with the life satisfaction rating.

## Discussion

Based on the large-scale CHARLS database, this study is the first to use interpretable ML methods to predict depressive symptoms in middle-aged and elderly individuals with malignant tumors. Six ML models, including XGBoost, DT, MLP, KNN, RF, and SVM, were developed and the decision-making abilities of these six algorithms were evaluated. The results showed that the RF model exhibited excellent predictive performance. The detailed performance indicators of the model were as follows: the AUC (95% CI) was 0.774 (0.740–0.809), the sensitivity was 0.802, the specificity was 0.828, and the accuracy (95% CI) was 0.724 (0.690–0.756). In fact, when applied to clinical diagnosis, when AUC > 0.7, the model can better distinguish between sick and non-ill individuals, indicating that the model is reliable [22]. SHAP further explained that the five most important features contributing to depression were life satisfaction, IADL score, self-rated health, pain, and average sleep duration. This will provide valuable insights for the early identification of depressive symptoms in clinical practice.

Using LASSO regression, we predicted 10 important variables. SHAP interpretability analysis showed, difficulty in activity, pain, female gender, and other types of comorbidities may be potential risk factors for depression. In contrast, higher life satisfaction, good health, longer average sleep duration, higher education level, participation in social activities, male gender and married status may avoid the risk of suffering from depression. The relationship between these factors and depression has been widely recognized in previous studies.

Negative self-rated health reflects the individual’s negative understanding of their own health status. This cognitive bias can easily lead to helplessness, weaken the individual’s ability to cope with stress, and make the individual more likely to fall into depression [23]. Impaired IADL function directly impacts the individual’s life autonomy, resulting in a decrease in self-worth, which may lead to doubts about their own ability and meaning of life, and further aggravate depressive symptoms [24]. Regarding gender differences in depression, Wang et al.’s study showed that the risk of depression in women with cancer is much higher than that in men [25]. The reasons for this outcome may be attributed to physiological factors such as the metabolic disorders of body hormones during women’s menopause, as well as the image damage they often face during the treatment process (such as surgery, chemotherapy, and radiotherapy) [26]. The long-term interaction of the two can easily lead to depression and seriously affect the treatment results. The waning of treatment effect will then react to mental health, resulting in a vicious circle.

Cancer with other types of comorbidities is very common. However, these comorbidities can often lead to pain and seriously affect the quality of life, making it difficult for them to remain optimistic about life in the long term [27, 28]. Poor personal health is often accompanied by chronic diseases, pain or discomfort. Such health problems can cause depression or social fear, which in turn increases the risk of depression [29]. These findings are also consistent with our research. Similarly, facing disability and chronic pain is associated with depression, which often leads to limitations in daily life, and these people often experience great psychological stress. In the long run, they may gradually lose confidence in their future life [30]. Studies have shown that sleep disorders are usually significantly associated with the occurrence of depressive symptoms [31]. Negative emotions such as anxiety, dysphoria and irritability are more common in groups with poor sleep quality, and the mechanism may be bidirectionally related. For example, sleep disorders can lead to depression, and vice versa [32]. Insomnia patients have increased activation or insufficient inhibition of the noradrenergic system, which is crucial for the processing and consolidation of emotional memory [33]. Orexin / hypothalamic secretin also plays an indispensable role in emotion regulation. Increased levels of orexin A in the cerebrospinal fluid of some patients with depression may lead to overactivation of the orexin system, which exacerbates insomnia by regulating the sleep-wake cycle [34].

It is gratifying that not all features are risk factors. People with high education levels may be more likely to obtain stronger self-adaptability and superior social resources, thus helping them to get rid of depression more easily [35]. In addition, participation in social activities is considered beneficial, and frequent social activities can promote interpersonal interaction and generate social identity, effectively reducing the risk of emotional abnormalities [36]. Similarly, the role of spouse is also crucial. Spouses are often better able to identify and alleviate patients’ emotional distress in a timely manner, thus helping patients cope with their daily lives more easily [37].

This study has the following advantages: Firstly, the CHARLS database used is a high-quality large-scale database from China, making the model more accurate. Secondly, LASSO regression only screened 10 key features, which are easy to obtain and have wide clinical applicability. Lastly, the interpretable model based on SHAP enables users to intuitively understand its mechanism for predicting the risk of depression in middle-aged and elderly cancer patients in China. Similar to other studies, our work also has some limitations. First, it must be admitted that this study lacks an external validation set. Second, although the data come from large databases, the sample size actually included in the study is small, and there may be significant regional bias in the database (for example, the sample size from developed provinces may be more than that from remote provinces). Third, the data set used in this study only includes Chinese patients, and whether the model is applicable to non-Chinese individuals needs further verification. Finally, as a retrospective study, there are still deficiencies compared to other methods such as prospective studies.

In view of the findings and limitations of this study, the following future exploration directions can be proposed. First of all, future research can enhance the universality of the model by recruiting larger and more diverse queues. Secondly, although the current ML models show effective prediction performance, the prediction performance will be further enhanced by applying more advanced deep learning models, such as recurrent neural networks (RNNs) or Transformer model. Finally, future research can focus on verifying the clinical practicability of the RF model in the real world through randomized controlled trials (RCT).

At the same time, if feasible, a dedicated website integrating the model can be constructed in the future, and the depression risk of middle-aged and elderly cancer patients can be reliably predicted by inputting the obtained survey indicators at the grassroots follow-up.

## Conclusion

In conclusion, this study used the prediction model constructed by ML algorithm to predict depression in middle-aged and elderly cancer patients in China. Among them, the RF model demonstrated high accuracy, robustness, and reliability. SHAP showed that the five key characteristics affecting the occurrence of depression were life satisfaction, instrumental activities of daily living score, self-rated health status, pain degree and average sleep duration. The SHAP-based interpretable predictive model we created has played an important clinical utility and may serve as a powerful tool for health care personnel to efficiently assess the risk of depression in cancer patients and provide individualized interventions.Future studies can design larger prospective RCT to further verify the applicability of the RF model in clinical applications.

## Electronic supplementary material

Below is the link to the electronic supplementary material.

Supplementary Material 1

## Data Availability

The data this study used can be accessed and downloaded from the following website: https://charls.charlsdata.com.

## Abbreviations

ML: machine learning
SHAP: Shapley Additive exPlanation
CHARLS: China Health and Retirement Longitudinal Study
LASSO: The least absolute shrinkage and selection operator
SVM: Support Vector Machine
KNN: K-Nearest Neighbor
DT: Decision Tree
RF: Random Forest
MLP: Multilayer Perceptron
XGBoost: EXtreme Gradient Boosting

## References

[1] Hanahan D, Weinberg RA. Hallmarks of cancer: the next generation. Cell. 2011;144(5):646–74. 21376230 10.1016/j.cell.2011.02.013

[2] Sung H, Ferlay J, Siegel RL, Laversanne M, Soerjomataram I, Jemal A, et al. Global Cancer statistics 2020: GLOBOCAN estimates of incidence and mortality worldwide for 36 cancers in 185 countries. Cancer J Clin. 2021;71(3):209–49. 10.3322/caac.21660 33538338

[3] Ding X, Wu M, Zhang Y, Liu Y, Han Y, Wang G, et al. The prevalence of depression and suicidal ideation among cancer patients in Mainland China and its provinces, 1994–2021: A systematic review and meta-analysis of 201 cross-sectional studies. J Affect Disord. 2023;323:482–9. 36496103 10.1016/j.jad.2022.12.011

[4] Zhao L, Li X, Zhang Z, Song C, Guo C, Zhang Y, et al. Prevalence, correlates and recognition of depression in Chinese inpatients with cancer. Gen Hosp Psychiatry. 2014;36(5):477–82. 24961793 10.1016/j.genhosppsych.2014.05.005

[5] Bota PJ, Wang C, Fred ALN, Silva HPDA, Review. Current challenges, and future possibilities on emotion recognition using machine learning and physiological signals. IEEE Access. 2019;7:140990–1020.

[6] Mahesh B. Machine Learning Algorithms -A Review2019.

[7] Esteva A, Kuprel B, Novoa RA, Ko J, Swetter SM, Blau HM, et al. Dermatologist-level classification of skin cancer with deep neural networks. Nature. 2017;542(7639):115–8. 28117445 10.1038/nature21056 PMC8382232

[8] Li S, Shi J, Shao C, Sznajder KK, Wu H, Yang X. Predicting depression, anxiety, and their comorbidity among patients with breast Cancer in China using machine learning: A multisite Cross-Sectional study. Depress Anxiety. 2024;2024:3923160. 40226665 10.1155/2024/3923160 PMC11918714

[9] Qamar T, Bawany NZ. Understanding the black-box: towards interpretable and reliable deep learning models. PeerJ Comput Sci. 2023;9:e1629. 38077598 10.7717/peerj-cs.1629 PMC10702969

[10] Nohara Y, Matsumoto K, Soejima H, Nakashima N. Explanation of Machine Learning Models Using Improved Shapley Additive Explanation. Proceedings of the 10th ACM International Conference on Bioinformatics, Computational Biology and Health Informatics; Niagara Falls, NY, USA: Association for Computing Machinery; 2019. p. 546.

[11] Zhao Y, Hu Y, Smith JP, Strauss J, Yang G. Cohort profile: the China health and retirement longitudinal study (CHARLS). Int J Epidemiol. 2014;43(1):61–8. 23243115 10.1093/ije/dys203 PMC3937970

[12] Zhao X, Wang Y, Li J, Liu W, Yang Y, Qiao Y, et al. A machine-learning-derived online prediction model for depression risk in COPD patients: A retrospective cohort study from CHARLS. J Affect Disord. 2025;377:284–93. 39988142 10.1016/j.jad.2025.02.063

[13] Wang N, Chang M, Liu S, Chen B. Study on the changes and influencing factors of depression in Chinese women with cancer: an analysis based on CHARLS panel data. Front Public Health. 2024;12:1485196. 39886394 10.3389/fpubh.2024.1485196 PMC11780894

[14] Tibshirani R. Regression shrinkage and selection via the Lasso. J Roy Stat Soc: Ser B (Methodol). 2018;58(1):267–88.

[15] Chen H, Mui AC. Factorial validity of the center for epidemiologic studies depression scale short form in older population in China. Int Psychogeriatr. 2014;26(1):49–57. 24125553 10.1017/S1041610213001701

[16] Burges CJC. A tutorial on support vector machines for pattern recognition. Data Min Knowl Disc. 1998;2(2):121–67.

[17] Nasteski V. An overview of the supervised machine learning methods. HORIZONSB. 2017;4:51–62.

[18] Song YY, Lu Y. Decision tree methods: applications for classification and prediction. Shanghai Archives Psychiatry. 2015;27(2):130–5. 10.11919/j.issn.1002-0829.215044 PMC4466856 26120265

[19] Breiman L. Random forests. Mach Learn. 2001;45(1):5–32.

[20] Gardner MW, Dorling SR. Artificial neural networks (the multilayer perceptron)—a review of applications in the atmospheric sciences. Atmos Environ. 1998;32(14):2627–36.

[21] Chen T, Guestrin C, XGBoost:. A Scalable Tree Boosting System. Proceedings of the 22nd ACM SIGKDD International Conference on Knowledge Discovery and Data Mining; San Francisco, California, USA: Association for Computing Machinery; 2016. pp. 785–94.

[22] Tantai XX, Liu N, Yang LB, Wei ZC, Xiao CL, Song YH, et al. Prognostic value of risk scoring systems for cirrhotic patients with variceal bleeding. World J Gastroenterol. 2019;25(45):6668–80. 31832005 10.3748/wjg.v25.i45.6668 PMC6906204

[23] Nieto I, Robles E, Vazquez C. Self-reported cognitive biases in depression: A meta-analysis. Clin Psychol Rev. 2020;82:101934. 33137610 10.1016/j.cpr.2020.101934

[24] Kiosses DN, Alexopoulos GS. IADL functions, cognitive deficits, and severity of depression: a preliminary study. Am J Geriatric Psychiatry: Official J Am Association Geriatric Psychiatry. 2005;13(3):244–9. 10.1176/appi.ajgp.13.3.244 15728756

[25] Li XL, Lin GY, Li KQ, Zhu LJ, Xu LW, Li SN. A meta-analysis on the incidence rate of depression in Chinese menopausal women. BMC Psychiatry. 2025;25(1):154. 39972281 10.1186/s12888-025-06603-y PMC11841152

[26] Holmes C, Alexis J, Joan L, Kasia G, Blakely K. Breast Cancer and body image: feminist therapy principles and interventions. J Feminist Family Therapy. 2021;33(1):20–39.

[27] Hawker GA, Gignac MA, Badley E, Davis AM, French MR, Li Y, et al. A longitudinal study to explain the pain-depression link in older adults with osteoarthritis. Arthritis Care Res. 2011;63(10):1382–90. 10.1002/acr.20298 20662042

[28] Ma Y, Xiang Q, Yan C, Liao H, Wang J. Relationship between chronic diseases and depression: the mediating effect of pain. BMC Psychiatry. 2021;21(1):436. 34488696 10.1186/s12888-021-03428-3 PMC8419946

[29] Read JR, Sharpe L, Modini M, Dear BF. Multimorbidity and depression: A systematic review and meta-analysis. J Affect Disord. 2017;221:36–46. 28628766 10.1016/j.jad.2017.06.009

[30] Bair MJ, Robinson RL, Katon W, Kroenke K. Depression and pain comorbidity: a literature review. Arch Intern Med. 2003;163(20):2433–45. 14609780 10.1001/archinte.163.20.2433

[31] Palmer CA, Bower JL, Cho KW, Clementi MA, Lau S, Oosterhoff B, et al. Sleep loss and emotion: A systematic review and meta-analysis of over 50 years of experimental research. Psychol Bull. 2024;150(4):440–63. 38127505 10.1037/bul0000410

[32] Yasugaki S, Okamura H, Kaneko A, Hayashi Y. Bidirectional relationship between sleep and depression. Neurosci Res. 2025;211:57–64. 37116584 10.1016/j.neures.2023.04.006

[33] Wassing R, Lakbila-Kamal O, Ramautar JR, Stoffers D, Schalkwijk F, Van Someren EJW, Restless. REM Sleep Impedes Overnight Amygdala Adaptation Curr Biology: CB. 2019;29(14):2351–e84. 10.1016/j.cub.2019.06.034 31303489

[34] Blouin AM, Fried I, Wilson CL, Staba RJ, Behnke EJ, Lam HA et al. Human hypocretin and melanin-concentrating hormone levels are linked to emotion and social interaction. 2013;4(1):1547. 10.1038/ncomms2461 PMC3595130 23462990

[35] Andersen BL, Lacchetti C, Ashing K, Berek JS, Berman BS, Bolte S, et al. Management of anxiety and depression in adult survivors of cancer: ASCO guideline update. J Clin Oncology: Official J Am Soc Clin Oncol. 2023;41(18):3426–53. 10.1200/JCO.23.00293 37075262

[36] Du M, Dai W, Liu J, Tao J. Less social participation is associated with a higher risk of depressive symptoms among Chinese older adults: A Community-Based longitudinal prospective cohort study. Front Public Health. 2022;10:781771. 35223728 10.3389/fpubh.2022.781771 PMC8863664

[37] Choi NG, Ha JH. Relationship between spouse/partner support and depressive symptoms in older adults: gender difference. Aging Ment Health. 2011;15(3):307–17. 21140305 10.1080/13607863.2010.513042 PMC3608851

