# Supplementary material for: An interpretable machine learning model for predicting depression in middle-aged and elderly cancer patients in China: a study based on the CHARLS cohort
Source: BMC Psychiatry. 2025 Jul 1;25:610. doi: 10.1186/s12888-025-07074-x (PMC12210965; doi:10.1186/s12888-025-07074-x)
Supplement: Supplementary file 1 — Supplementary Material 1 [file 12888_2025_7074_MOESM1_ESM.docx]

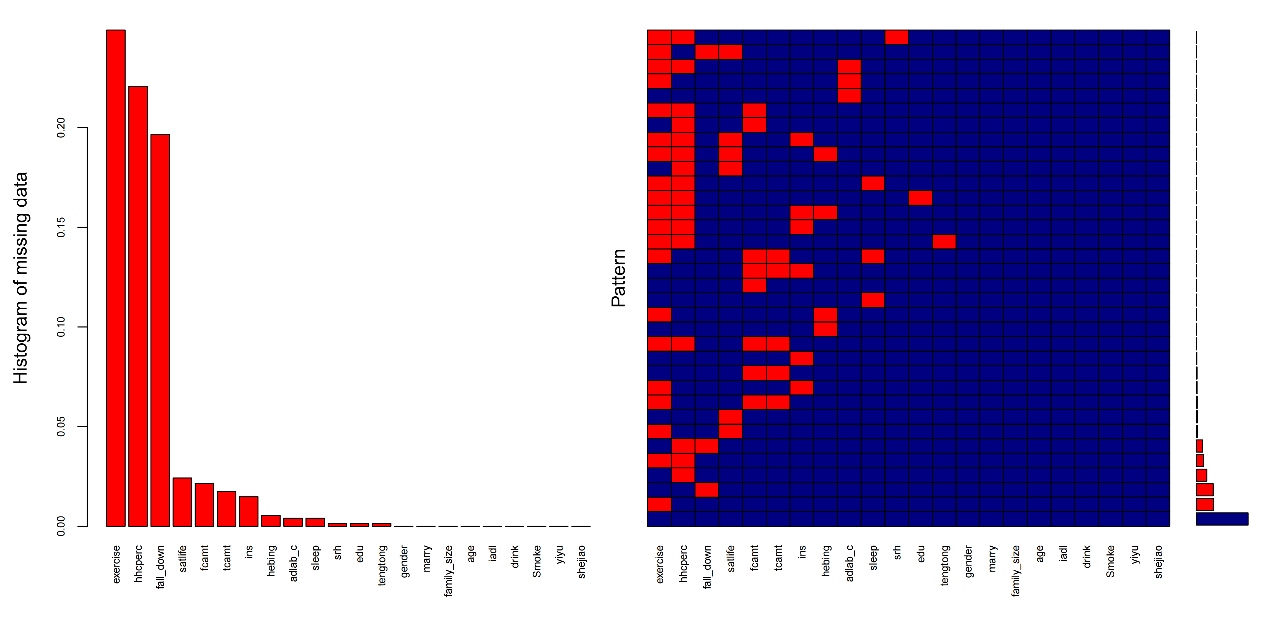


**Supplementary Figure 1. Missing data structure.**


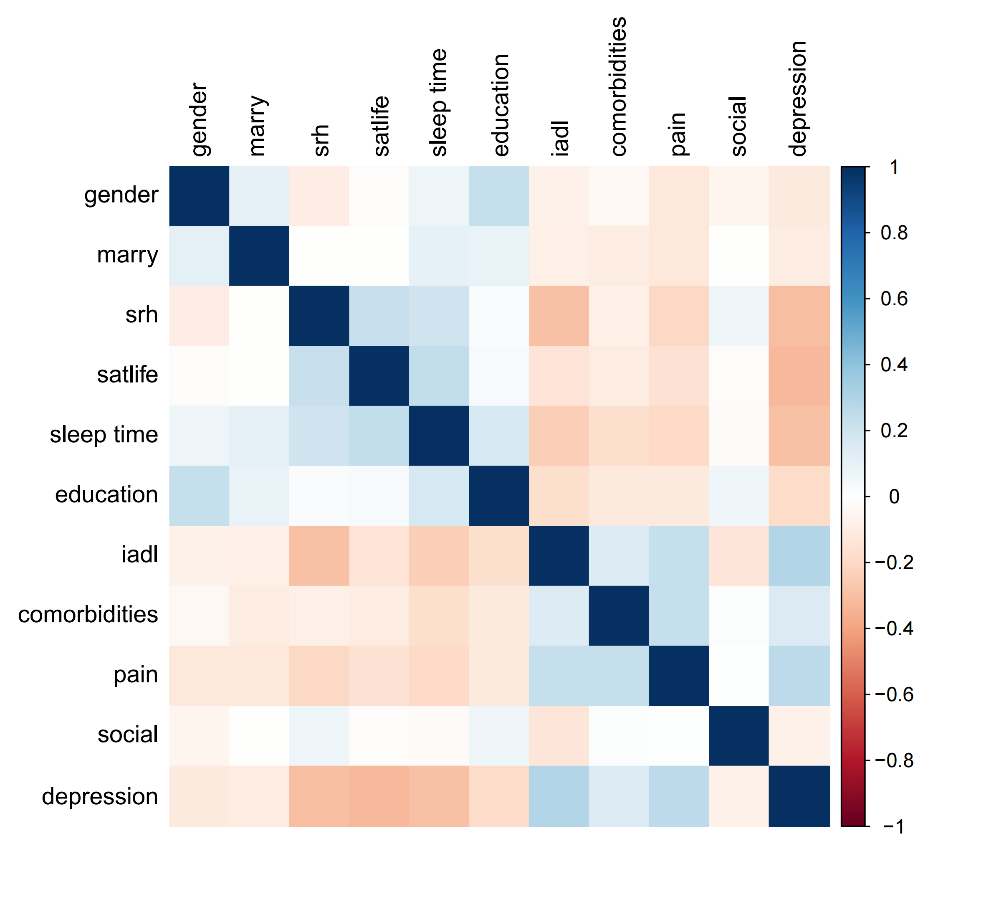


**Supplementary Figure 2. Correlation matrix heatmap of variables.** Blue represents positive correlation, and red represents negative correlation. The darker the color, the stronger the correlation between variables.
